# Classification and prediction of cognitive trajectories of cognitively unimpaired individuals

**DOI:** 10.3389/fnagi.2023.1122927

**Published:** 2023-03-13

**Authors:** Young Ju Kim, Si Eun Kim, Alice Hahn, Hyemin Jang, Jun Pyo Kim, Hee Jin Kim, Duk L. Na, Juhee Chin, Sang Won Seo

**Affiliations:** ^1^Department of Neurology, Samsung Medical Center, Sungkyunkwan University School of Medicine, Seoul, Republic of Korea; ^2^Neuroscience Center, Samsung Medical Center, Seoul, Republic of Korea; ^3^Department of Neurology, Haeundae Paik Hospital, Inje University College of Medicine, Busan, Republic of Korea; ^4^Department of Mental Health, Johns Hopkins Bloomberg School of Public Health, Baltimore, MD, United States; ^5^Center for Neuroimaging, Radiology and Imaging Sciences, Indiana University School of Medicine, Indianapolis, IN, United States; ^6^Institute of Stem Cell and Regenerative Medicine, Seoul, Republic of Korea; ^7^Samsung Alzheimer Research Center, Samsung Medical Center, Seoul, Republic of Korea; ^8^Center for Clinical Epidemiology, Samsung Medical Center, Seoul, Republic of Korea; ^9^Department of Health Sciences and Technology, Seoul, Republic of Korea; ^10^Clinical Research Design and Evaluation, Samsung Advanced Institute for Health Sciences & Technology (SAIHST), Sungkyunkwan University, Seoul, Republic of Korea

**Keywords:** cognitive trajectory, cognitively unimpaired, nomogram, prediction, classification

## Abstract

**Objectives:**

Efforts to prevent Alzheimer’s disease (AD) would benefit from identifying cognitively unimpaired (CU) individuals who are liable to progress to cognitive impairment. Therefore, we aimed to develop a model to predict cognitive decline among CU individuals in two independent cohorts.

**Methods:**

A total of 407 CU individuals from the Alzheimer’s Disease Neuroimaging Initiative (ADNI) and 285 CU individuals from the Samsung Medical Center (SMC) were recruited in this study. We assessed cognitive outcomes by using neuropsychological composite scores in the ADNI and SMC cohorts. We performed latent growth mixture modeling and developed the predictive model.

**Results:**

Growth mixture modeling identified 13.8 and 13.0% of CU individuals in the ADNI and SMC cohorts, respectively, as the “declining group.” In the ADNI cohort, multivariable logistic regression modeling showed that increased amyloid-β (Aβ) uptake (β [SE]: 4.852 [0.862], *p* < 0.001), low baseline cognitive composite scores (β [SE]: −0.274 [0.070], *p* < 0.001), and reduced hippocampal volume (β [SE]: −0.952 [0.302], *p* = 0.002) were predictive of cognitive decline. In the SMC cohort, increased Aβ uptake (β [SE]: 2.007 [0.549], *p* < 0.001) and low baseline cognitive composite scores (β [SE]: −4.464 [0.758], *p* < 0.001) predicted cognitive decline. Finally, predictive models of cognitive decline showed good to excellent discrimination and calibration capabilities (C-statistic = 0.85 for the ADNI model and 0.94 for the SMC model).

**Conclusion:**

Our study provides novel insights into the cognitive trajectories of CU individuals. Furthermore, the predictive model can facilitate the classification of CU individuals in future primary prevention trials.

## Introduction

Populations around the world are aging more rapidly, and aging-related health issues and diseases are projected to lead to greater societal and economic burdens (Patterson, 2018). As individuals age, they may experience progressive physiological (“normal”) cognitive decline, although each follows a unique trajectory (Deary et al., 2009). In particular, reductions in memory, conceptual reasoning, and processing speed are frequently observed in aged individuals (Committee on the Public Health Dimensions of Cognitive Aging et al., 2015). Pathological cognitive decline may occur due to Alzheimer’s disease (AD)-related processes, eventually resulting in mild cognitive impairment (MCI) due to AD or AD dementia (Mormino and Papp, 2018). The WHO reported in 2012 that about 150 million people will be impacted by dementia by 2050, with a consequent increase in the total costs of AD to over $1 trillion (Patterson, 2018). Therefore, early diagnosis and prevention are critical to reduce the burdens of AD. In particular, AD prevention may be improved by identifying cognitively unimpaired (CU) individuals who are liable to progress to cognitive impairment (Jack et al., 2018).

Previous studies of early AD diagnosis were based on hypothesis-driven analyses, in which researchers classified individuals into subgroups based on their hypothesis. In contrast, in trajectory analyses, individuals are classified into distinct subgroups or classes using a data-driven classification method (Nguena Nguefack et al., 2020). Trajectory analyses enable researchers to better characterize and understand intra- and inter-individual variability as well as to investigate the patterns of health outcomes in longitudinal data (Jung and Wickrama, 2008). In this regard, mixture modeling approaches, such as growth mixture modeling (GMM) and latent class growth analysis (LCGA), have been increasingly used to identify homogeneous subpopulations within a larger heterogeneous population and to identify meaningful classes (Jung and Wickrama, 2008). Group-based models have been applied to patients with cognitive impairments in order to identify developmental trajectories (David et al., 2016; Lee et al., 2018; Kim S. J. et al., 2022). However, there have been only a few studies applying trajectory analyses to CU individuals (Small and Bäckman, 2007; Min, 2018).

Previously, several factors have been found to influence the progression of CU to MCI or dementia (Rusinek et al., 2003; Chen et al., 2017; Mormino and Papp, 2018; Cho H. et al., 2020). Specifically, advanced age, reductions in baseline executive function, and smaller total brain volume were independently associated with the risk of conversion to MCI (Chen et al., 2017). In addition, CU individuals with the apolipoprotein E ε4 allele (*APOE* ε4), cerebral amyloid burdens, and cortical atrophy were more likely to progress to cognitive impairment (Rusinek et al., 2003; Mormino and Papp, 2018; Cho H. et al., 2020). While these study results are important, methods to translate such findings to clinical practice are needed in order to enable individualized prediction of cognitive decline.

As a prediction model for personalized application, the nomogram is a valuable tool. A nomogram is a chart describing the numerical relationships between diseases and risk factors and their graphical calculation (Kattan and Marasco, 2010). Based on the specific characteristics of patients or diseases, it is designed to help doctors and patients for risk assessment and predicting results of treatment (Tsikitis et al., 2016). Also, with its advantages of visual presentation and easy accessibility, nomogram can be easily used in busy clinical environments (Kim W. et al., 2016). Nomograms have been applied for more than a decade in oncology or cardiology. Nomograms are being developed and used more frequently in patients with cognitive deficits (Jang et al., 2017; Kim et al., 2018), but their development and application are lacking for CU individuals.

In the present study, we aimed to investigate the feasibilities of classification of cognitive trajectories of CU individuals in two independent cohorts with different genetic and sociocultural backgrounds. First, we determined if there were distinct growth patterns in the cognitive trajectories of the CU individuals by using mixture modeling in two independent cohorts. Second, we evaluated the features that significantly impact the classification of latent class. We hypothesized that there might be differences in the effects of features on the classification between the two datasets because participants of the two datasets have different genetic and sociocultural backgrounds. Finally, using these features, we developed a predictive model for cognitive decline in CU individuals and a nomogram to visualize risk probability.

## Materials and methods

### Clinical data collection

We collected the data from two independent cohorts. Data used for the preparation of the current study were obtained from the ADNIMERGE dataset of the Alzheimer’s Disease Neuroimaging Initiative (ADNI) database (adni.loni.usc.edu) (*n* = 407) and from the Samsung Medical Center (SMC) (*n* = 285).

The ADNI was launched in 2003 as a public-private partnership, led by principal investigator Michael W. Weiner, MD. The primary purpose of the ADNI is to test whether serial magnetic resonance imaging (MRI), positron emission tomography (PET), other biological markers, and clinical or neuropsychological assessments, can be combined to evaluate the progression of MCI and early AD. For up-to-date information, see www.adni-info.org. Participants from the ADNI-1 to ADNI-3 and ADNI GO were included in the current study if (1) their baseline diagnosis was CU or subjective memory complaints, (2) they had valid cognitive assessments, and (3) they had at least two follow-up assessments of cognitive function. The baseline diagnoses were determined using the standard criteria described in the ADNI procedure manuals.^1^ As a result, a total of 407 individuals met these qualifications to be included in the current study. All individuals had usable neuropsychological data, and 352 individuals had hippocampal volumetric data.

In addition, 285 CU individuals were recruited from the SMC. These patients also had usable neuropsychological data, and 231 individuals had hippocampal volumetric data. They underwent amyloid PET scans at the SMC between September 2015 and December 2021, and were followed up at least twice through clinical interviews and thorough cognitive tests until December 2021. The following were used to establish the baseline diagnostic criteria for CU: (i) the Korean Mini-Mental State Examination (K-MMSE) ≥24 or above −1.5 SD from the age-, gender-, and education-adjusted norms if education level was <9 years; (ii) above −1 SD from the age-, gender-, and education-adjusted norms on the delayed recall of the Seoul Verbal Learning Test-Individuals version (SVLT-E); (iii) above −2 SD from the age-, gender-, and education-adjusted norms on the Korean version of the Boston Naming Test (K-BNT), the Rey-Osterrieth Complex Figure Test (RCFT) copy, and the Korean Color Word Stroop Test (K-CWST) color reading; and (iv) absence of other neurological diseases.

### Standard protocol approval, registration, and patient consent

The authors obtained approval from the ADNI Data Sharing and Publications Committee for data use and publication. No Institutional Review Board (IRB) review approval was required to use de-identified ADNI data that is available for download. All methods were carried out in accordance with the approved guidelines. At the SMC, the IRB approved the use of SMC data for this study, and all of the methods used were carried out in compliance with the approved standards.

### Acquisition of neuroimaging data

The current study employed the following neuroimaging data from the ADNIMERGE dataset: average AV45 standardized uptake value ratios (SUVRs) of the frontal cortex, parietal cortex, anterior cingulate cortex and precuneus relative to the cerebellum, and the hippocampal volume (HV). The detailed protocols for image acquisition have been described in previous studies (Jagust et al., 2010) and the ADNI database.^2^ Cortical reconstruction and volumetric segmentation were performed using the FreeSurfer image analysis program (Hartig et al., 2014).^3^

For SMC data, all individuals underwent either FBB or FMM PET scans at the SMC using a Discovery STe PET/CT scanner (GE Medical Systems, Milwaukee, WI, USA) in 3D scanning mode to examine 47 slices of 3.3-mm thickness spanning the entire brain (Kim et al., 2018; Jang et al., 2019). In our previous study, we used a direct comparison Centiloid units (dcCL) conversion equation to directly convert the SUVR values of the FBB or FMM cortical target volume of interest (CTX VOI) into dcCL units (Klunk et al., 2015; Cho S. H. et al., 2020). The conversion was performed using equations for the FBB (dcCL_FBB_ = 151.42 × dcSUVR_FBB_ − 142.24); and the FMM (dcCL_FMM_ = 148.52 × dcSUVR_FMM_ − 137.09) PET. All individuals also underwent 3D T1-weighted turbo field echo MRI at SMC using a 3.0-T MRI scanner (Philips 3.0T Achieva; Philips Healthcare, Andover, MA, USA) as previously described (Kim H. J. et al., 2016). Images were processed using the CIVET anatomical image-processing pipeline (version 2.1.0) (Zijdenbos et al., 2002). We calculated the intracranial volume (ICV) by measuring the total volume of the voxels within the brain mask (Smith, 2002). The FMRIB (Functional Magnetic Resonance Imaging of the Brain) Software Library (FSL) method was used to create brain masks. Since cortical surface models were extracted from brain MRI volumes transformed into stereotaxic space, cortical thickness was assessed in the native space by applying an inverse transformation matrix to the cortical surfaces and rebuilding them in native space (Im et al., 2006). To measure HV, we employed a computerized hippocampus segmentation technique that combined graph-cut optimization, atlas-based segmentation, and morphological opening (Kwak et al., 2013).

### Neuropsychological assessments

The current study used the ADNI-modified Preclinical Alzheimer Cognitive Composite with the Trail-Making Test, Part B time to completion (mPACCtrt) as the cognitive endpoint from the original ADNIMERGE dataset. The PACC was developed as an outcome measure of cognitive changes in preclinical AD (Donohue et al., 2014). The original version includes (a) the total recall score of the Free and Cued Selective Reminding Test (FCSRT), (b) the delayed recall score of the Logical Memory IIa (LM), (c) the Digit Symbol Substitution score from the Wechsler Adult Intelligence Scale revised version (DSST), (d) Mini-Mental State Examination (MMSE) total score. Because the FCSRT was not included in the ADNI battery, the FCSRT was replaced by the delayed recall of the Alzheimer’s Disease Assessment Scale (ADAS) in the mPACCtrt. Also, we used the Trail-Making Test, Part B (TMT-B) instead of the DSST (Donohue et al., 2014). As a result, the mPACCtrt consisted of (a) ADAS-cognitive subscale delayed word recall, (b) logical memory delayed recall, (c) the MMSE total score, and (d) (log-transformed) trail-making test time to completion. The composite score was the sum of the *z*-scores of each constituent test, which were based upon the mean and standard deviations of the baseline scores of the CU individuals in the ADNI (Donohue et al., 2014, 2017).

For the SMC dataset, the Longitudinal Amyloid Cognitive Composite in Preclinical AD (LACPA) was used. The LACPA was developed for longitudinal tracking of amyloid-β (Aβ)-related cognitive decline in CU individuals using well-characterized and relatively large Korean CU cohorts (Kim Y. J. et al., 2022). The LACPA equation is as follows:


LACPA=SVLT⁢IR⁢Z+SVLT⁢DR⁢Z+SVLT⁢recognition⁢Z

+K-TMT-B⁢time⁢Z+K-MMSE⁢Z/Number⁢of⁢tests

where IR is the immediate recall, DR is the delayed recall, K-TMT-B is the Korean Trail-Making Test Part B and Z is the *z*-score.

### Statistical analyses

We used mixture modeling to test for distinct growth patterns in the cognitive trajectories of the CU individuals. Mixture modeling generally uses categorical latent variables representing the composition of a subpopulation, in which case the members of the subpopulation are unknown and inferred from the data (Wang and Bodner, 2007). In mixture modeling using longitudinal data, unperceived heterogeneity is captured through categorical and continuous latent variables (Wang and Bodner, 2007). We used mixture modeling with GMM and the LCGA for the ADNI dataset and SMC datasets, respectively. The LCGA is a special type of GMM that limits the variance and covariance estimates for growth factors within each class to zero (Jung and Wickrama, 2008). To determine the adequate number of latent classes, we compared the following four methods: Bayesian information criterion (BIC) (Schwarz, 1978); Akaike information criterion (AIC) (Akaike, 1974); Lo, Mendell, Rubin test (LMR) (Lo et al., 2001); and parametric bootstrapped likelihood ratio test (BLRT) (McLachlan and Peel, 2000). After determining the number of latent classes, the baseline characteristics of the latent classes were analyzed using the independent *t*-test and the Chi-square test.

**FIGURE 1:**
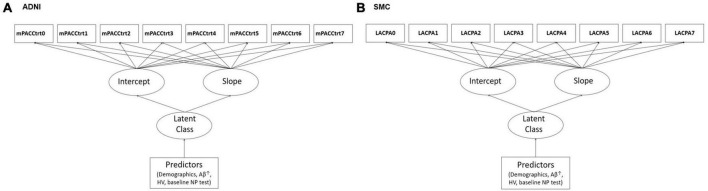
Illustrations of the study models including predictors in the latent growth mixture modeling in **(A)** ADNI and **(B)** SMC. ^↑^AV45 SUVR in ADNI and dcCL in SMC. ADNI, Alzheimer’s Disease Neuroimaging Initiative; SMC, Samsung Medical Center; mPACCtrt, modified Preclinical Alzheimer’s Cognitive Composite with Trail-Making Test, Part B; LACPA, Longitudinal Amyloid Cognitive Composite in Preclinical AD; HV, hippocampal volume; NP, neuropsychological.

To investigate the effects of variables on the cognitive trajectory group based on the mPACCtrt score derived from the ADNI dataset, we performed multivariable logistic regression analyses, which include AV45 SUVR, baseline mPACCtrt score, and HV associated with covariates including age, gender, education level, *APOE*ε4. In addition, to determine the effects of variables on the cognitive trajectory group based on the LACPA score derived from the SMC dataset, we conducted multivariable logistic regression analyses, which include dcCL, baseline LACPA score, and HV associated with covariates including age, gender, education level, and *APOE* ε4 (Figure 1). The HV was used as the value divided by the ICV (HV/ICV) to consider the cranial cavity. The corrected HV was multiplied by 1,000, because of the relatively small scale. The dcCL was used as the value divided by 100.

After developing the predictive models, we constructed a nomogram using the dataset. We assigned a point value to each variable using the beta coefficients from the logistic regression model. Then, the most powerful variable was set at 100 points, while other variables were assigned between 0 and 100 points based on their proportions. The total points summed by the scores corresponding to each variable were immediately converted to risk probability. Finally, the predictive accuracy of the nomogram was verified by discrimination (C-index) and calibration.

Mplus version 8.3 was used for the GMM, and the LCGA (Muthén and Muthén, 2019). Full information maximum likelihood (FIML) estimation was employed for the missing values found in the longitudinal data. R 4.0.3 (Vienna, Austria^4^) was used for the logistic regression analyses and to develop the nomogram.

### Data availability

The datasets used and/or analyzed during the present study are available from the corresponding authors on reasonable request.

## Results

### Demographic characteristics

The baseline demographic characteristics of the CU individuals included in the ADNI and SMC datasets are shown in Table 1. The mean age of the individuals was 73.2 years in the ADNI dataset and 70.7 years in the SMC dataset (*p* < 0.001). The proportion of *APOE* ε4 carriers was 30.2 and 29.8% in the ADNI dataset and the SMC dataset, respectively. The proportion of females was 53.8% in the ADNI dataset and 61.8% in the SMC dataset (*p* = 0.038).

**TABLE 1:** Baseline demographic and neuropsychological characteristics of the study participants.

Variables	ADNI (*n* = 407)	SMC (*n* = 285)	*p*-Value
Age, years	73.2 ± 6.1	70.7 ± 6.9	<0.001
Education, years	16.6 ± 2.6	11.7 ± 4.7	<0.001
Female, *N* (%)	219 (53.8)	176 (61.8)	0.038
*APOE*ε4 carrier *N* (%)	123 (30.2)	85 (29.8)	0.913

*N*, number; ADNI, Alzheimer’s Disease Neuroimaging Initiative; SMC, Samsung Medical Center; *APOE*ε4, apolipoprotein E ε4 allele.

### Identifying distinguishable trajectory subgroups

For the ADNI dataset, to explore the number of latent class, BIC, adjusted BIC and AIC values were compared as we increased the number of classes to determine the appropriate model. Then, we compared the models using the LMR and BLRT (Table 2). Although the three-class model exhibited smaller AIC and adjusted BIC values, the two-class model had the smallest BIC value. Moreover, the difference between these values when obtained from the two- and three-class models was minimal compared to the difference for the one- and two-class models. This finding indicated that the two-class model was an appropriate model. To determine whether the two- or three-class model was better, we also used the LMR and BLRT. The LMR comparing the two- and three-class models indicated the two-class model was better (*p* = 0.014), and the BLRT result also supported the two-class model (*p* < 0.001). Considering these results comprehensively, we decided that the two-class model would be adequate for the final model. Its entropy (the quality of the classification) was high at 0.79. For the SMC dataset, the LMR comparing the one-, two-, and three-class models indicated that the two-class model was better (*p* = 0.018), and the BLRT result also supported the two-class model (*p* < 0.001). Considering these results comprehensively, we again decided that the two-class model would be adequate for the final model. Its entropy, the quality of the classification, was high at 0.861.

**TABLE 2:** Fitting information for the growth mixture models of cognitive trajectories from the ADNI and SMC cohort datasets.

Model selection measures	ADNI			SMC		
	**One-class**	**Two-class**	**Three-class**	**One-class**	**Two-class**	**Three-class**
AIC	8,306.216	8,239.018	8,233.775	2,093.981	1,890.766	1,794.979
BIC	8,358.331	8,303.159	8,309.942	2,130.506	1,938.248	1,853.419
Adjusted BIC	8,317.080	8,252.389	8,249.653	2,098.795	1,897.025	1,802.682
LMR *p*-value	–	0.014	0.449	–	0.018	0.050
BLRT *p*-value	–	<0.001	0.066	–	<0.001	<0.001
Entropy	–	0.790	0.815	–	0.861	0.763

ADNI, Alzheimer’s Disease Neuroimaging Initiative; SMC, Samsung Medical Center; AIC, Akaike information criterion; BIC, Bayesian information criterion; LMR, Lo, Mendell, Rubin test; BLRT, parametric bootstrapped likelihood ratio test.

**FIGURE 2:**
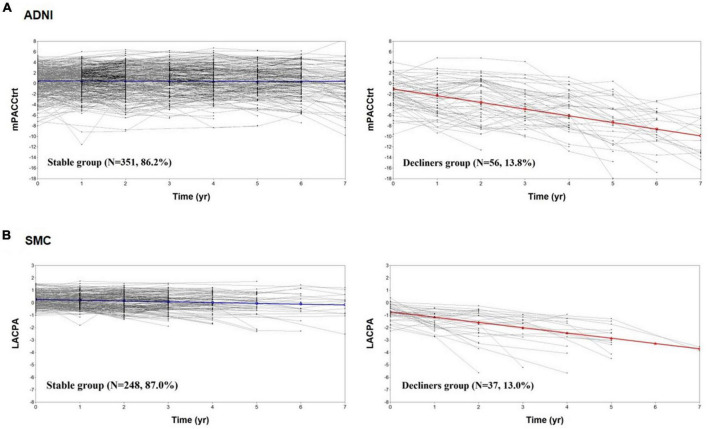
The cognitive trajectories of the two-class model. The mPACCtrt **(A)** and LACPA **(B)** for the two-class unconditional growth mixture modeling solution. ADNI, Alzheimer’s Disease Neuroimaging Initiative; SMC, Samsung Medical Center; mPACCtrt, modified Preclinical Alzheimer’s Cognitive Composite with Trail-Making Test, Part B; LACPA, Longitudinal Amyloid Cognitive Composite in Preclinical AD; *N*, number; yr, year.

Figure 2 shows the cognitive trajectories of the two-class model using the PACC and LACPA for the ADNI and SMC datasets, respectively. In the ADNI dataset, Class 1, in which the estimated class percentage was 86.2%, was a group whose cognitive function remained stable for 7 years (intercept = 0.479, SE = 0.148, *p* = 0.001; slope = −0.012, SE = 0.027, *p* = 0.649). Class 2, in which the estimated class percentage was 13.8%, showed cognitive decline during the same period (intercept = −1.058, SE = 0.534, *p* = 0.047; slope = −1.262, SE = 0.088, *p* < 0.001). Thus, we identified Class 1 a “stable group” and Class 2 as a “declining group” (Figure 2A). The same trajectory subgroups were also identified in CU individuals of the SMC dataset. Class 1, in which the estimated class percentage was 87.0%, was a group whose cognitive function remained stable for 7 years (intercept = 0.257, SE = 0.045, *p* < 0.001; slope = −0.060, SE = 0.023, *p* = 0.008). Class 2, in which the estimated class percentage was 13.0%, showed cognitive decline during the same period (intercept = −0.734, SE = 0.180, *p* < 0.001; slope = −0.424, SE = 0.075, *p* < 0.001) (Figure 2B).

### Comparisons of the clinical characteristics of the trajectory subgroups

The baseline characteristics of the two trajectory groups in the ADNI dataset (351 stable and 56 decliners), and the SMC dataset (248 stable and 37 decliners) are shown in Table 3. The data analyses indicated there were significant predictive factors among the demographic variables and biomarkers at baseline identifying individuals who were at risk of cognitive decline among the two cohorts. The mean age was higher in the decliners than in the stable groups of both datasets (*t*-test; ADNI *p* < 0.001; SMC *p* = 0.032). The years of education and gender distribution did not significantly differ between the two groups. The presence of *APOE*ε4 was more frequent among decliners than in the stable group (Chi-square test; ADNI *p* = 0.028; SMC *p* = 0.021). In a comparison of biomarkers including Aβ and HV, Aβ uptake was higher in the decliners than in the stable group (*t*-test; ADNI *p* < 0.001; SMC *p* < 0.001). In terms of the HV, it was lower in the decliners than in the stable group in ADNI, whereas there was no significant difference between the two groups in the SMC dataset (*t*-test; ADNI *p* < 0.001; SMC *p* = 0.063). The baseline neuropsychological composite score was lower in the decliners than in the stable group in both datasets (*t*-test; ADNI *p* < 0.001; SMC *p* < 0.001).

**TABLE 3:** Baseline demographic and neuropsychological characteristics of the individuals assigned to each latent class.

Variables	ADNI		SMC	
	**Stable (*N* = 351)**	**Decliners (*N* = 56)**	**Stable (*N* = 248)**	**Decliners (*N* = 37)**
Age, years^a^	72.7 (6.2)	75.7 (5.2)*	70.4 (7.0)	73.0 (6.0)*
Education, years^a^	16.6 (2.6)	16.5 (2.4)	11.6 (4.6)	12.2 (5.0)
Female, *N* (%)	189 (53.8)	30 (53.6)	152 (61.3)	24 (64.9)
*APOE*ε4 carrier *N* (%)	99 (28.3)	24 (42.9)*	68 (28.2)	17 (47.2)*
Aβ uptake^a↑^	1.1 (0.2)	1.3 (0.2)**	24.9 (39.0)	57.5 (48.7)**
HV/ICV^a^	5.0 × 10^–3^ (6.3 × 10^–4^)	4.6 × 10^–3^ (6.7 × 10^–4^)^**^	2.3 × 10^–3^ (3.2 × 10^–4^)	2.2 × 10^–3^ (2.9 × 10^–4^)
Baseline neuropsychological composite score^a↑↑^	0.36 (2.45)	−1.84 (2.81)**	0.25 (0.52)	^–^0.75 (0.62)**

*p < 0.05; **p < 0.001.

^a^Values are the mean and SD. ^↑^AV45 SUVR in ADNI and dcCL in SMC. ^↑↑^Modified Preclinical Alzheimer’s Cognitive Composite with Trail-Making Test, Part B in the ADNI cohort, Longitudinal Amyloid Cognitive Composite in Preclinical AD in the SMC cohort. ADNI, Alzheimer’s Disease Neuroimaging Initiative; SMC, Samsung Medical Center; *APOE*ε4, apolipoprotein E ε4 allele; Aβ, amyloid-β; SUVR, standardized uptake value ratio; dcCL, direct comparison Centiloid units; HV, hippocampal volume; ICV: intracranial volume.

### Development of the predictive model

Multivariable logistic regression was used to analyze the associations between cognitive decline and potential predictors. Table 4 shows the significant measures predicting cognitive decline. In the model using the ADNI dataset, the multivariable logistic regression model showed that Aβ uptake, baseline neuropsychological composite score and HV were predictive of being included in the cognitive decline group. Increased Aβ uptake (β [SE]: 4.852 [0.862], *p* < 0.001), lower baseline neuropsychological composite score (β [SE]: −0.274 [0.070], *p* < 0.001), and lower HV (β [SE]: −0.952 [0.302], *p* = 0.002) were significantly correlated with greater likelihood of being included in the cognitive decline group. The predictive performance (C-index) in the ADNI model was 0.85 (Table 4). In the model using the SMC dataset increased Aβ uptake (β [SE]: 2.007 [0.549], *p* = 0.007) and lower baseline neuropsychological composite scores (β [SE]: −4.464 [0.758] *p* < 0.001) were significantly correlated with greater risk probability. The C-index in the SMC model was 0.94. In addition, gender (β [SE]: 0.517 [0.386], *p* = 0.180) in ADNI data, age (β [SE]: 0.081 [0.039], *p* = 0.038) and gender (β [SE]: 1.093 [0.532], *p* = 0.040) in SMC data were also statistically significant variables when the *p*-value is based on 0.2, although the statistical significance is lower than other variables (Table 4).

**TABLE 4:** Multivariable logistic regression for the predictive model.

	Predictors	*B*	SE	OR	95% CI	*p*	C-index
ADNI	Intercept	-3.311	1.724	0.036	−	0.055	0.854
	Female	0.517	0.386	1.678	0.788, 3.574	0.180	
	Aβ uptake^↑^	4.852	0.862	128.013	23.628, 693.563	<0.001	
	HV/ICV	-0.952	0.302	0.386	0.214, 0.697	0.002	
	Baseline neuropsychological composite score^↑↑^	-0.274	0.070	0.760	0.663, 0.872	<0.001	
SMC	Intercept	-10.281	3.017	3.4 × 10^–5^	−	0.001	0.941
	Age	0.081	0.039	1.084	1.004, 1.171	0.038	
	Female	1.093	0.532	2.983	1.052, 8.464	0.040	
	Aβ uptake (CL)^↑^	2.007	0.549	7.438	2.536, 21.817	<0.001	
	Baseline neuropsychological composite score^↑↑^	-4.464	0.758	0.012	0.003, 0.051	<0.001	

^↑^AV45 SUVR in ADNI and dcCL in SMC. ^↑↑^Modified Preclinical Alzheimer’s Cognitive Composite with Trail-Making Test, Part B in ADNI cohort, Longitudinal Amyloid Cognitive Composite in Preclinical AD in SMC cohort. ADNI, Alzheimer’s Disease Neuroimaging Initiative; SMC, Samsung Medical Center; Aβ, amyloid-β; SUVR, standardized uptake value ratio; dcCL, direct comparison Centiloid units; HV, hippocampal volume; ICV, intracranial volume; SE, standard error; OR, odds ratio; 95% CI, 95% confidence interval.

**FIGURE 3:**
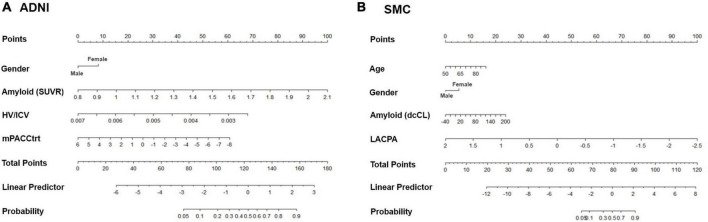
Nomograms predicting the cognitive decline of the cognitively unimpaired individuals in **(A)** ADNI and **(B)** SMC. Age (only in SMC), gender, amyloid uptake, hippocampal volume (only in ADNI), and baseline neuropsychological composite score are variables that significantly and independently affected the cognitive trajectory of the cognitively unimpaired individuals. Using odd ratios from the regression model, each variable was assigned points. The total points from all variables indicated the probability of the declining group in the nomogram. ADNI, Alzheimer’s Disease Neuroimaging Initiative; SMC, Samsung Medical Center; SUVR: standardized uptake value ratio; dcCL: direct comparison Centiloid units; HV, hippocampal volume; ICV, intracranial volume; mPACCtrt, modified Preclinical Alzheimer’s Cognitive Composite with Trail-Making Test, Part B; LACPA, Longitudinal Amyloid Cognitive Composite in Preclinical AD.

We then constructed nomograms based on the multivariable logistic regression results (Figure 3). The total points were calculated from the sum of each point, and the risk probability was calculated from the total points. The probability in our nomograms referred to risk probability of being assigned to a cognitive decline group. The bias-corrected calibrated values were generated from validations based on 1,000 bootstrap resamples. The non-parametric calibration curves revealed that the bias-corrected calibration plots were close to the 45° line, indicating the nomograms are well calibrated (Supplementary Figure 1).

### Visualization of the predictive model

**FIGURE 4:**
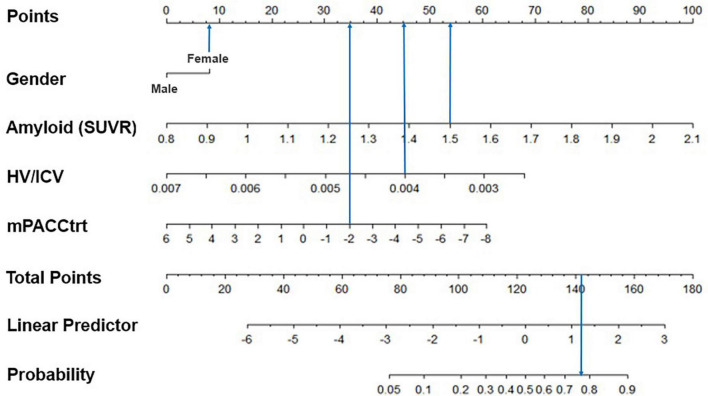
Example of the graphical representation of the model predictors. The subject was a woman with an AV45 SUVR of 1.5, HV/ICV of 0.004, and an mPACCtrt of –2. As a result, her total score was 142 and the model predicted her corresponding probability of cognitive decline would be 77.2%. SUVR, standardized uptake value ratio; HV, hippocampal volume; ICV, intracranial volume; mPACCtrt, modified Preclinical Alzheimer’s Cognitive Composite with Trail-Making Test, Part B.

Finally, we visualized the risk score of each predictor and the predicted probability of belonging to a trajectory group using the predictive model (Figure 4). Specifically, for a woman with an AV45 SUVR of 1.5, HV/ICV of 0.004 and an mPACCtrt of −2, the risk scores of all predictors are 142, so the probability of belonging to the declining group was estimated to be 77.2%.

## Discussion

In the present study, we explored the cognitive trajectories of CU individuals in two independent cohorts identified from the ADNI and SMC datasets. Our major findings were as follows. First, 13.8% of the CU individuals in the ADNI dataset were classified in the declining group, which was similar to the proportion of the decliners in the SMC dataset. Second, increased amyloid uptake, lower baseline neuropsychological composite score, and decreased HV (only in ADNI) were predictive of being classified within the declining group. Finally, predictive models of cognitive decline showed fair to good discrimination and calibration capabilities. Taken together, our analysis provided novel insights into the different cognitive trajectories of CU individuals. Furthermore, the predictive model may facilitate the classification of CU individuals, and could be employed in future primary prevention trials.

Our first major finding was that 13.8% of the CU individuals in the ADNI dataset were classified to the declining group, similar to the results for the SMC dataset. Regarding the conversion from CU to MCI, a previous study revealed that clinic-based sample populations demonstrated an annual conversion rate of 30% (95% CI 17–54%) per person-year, and the community-based sample population demonstrated a conversion rate of 5% (95% CI 3–6%) per person-year (Chen et al., 2017). In another study, 6.7% of CU individuals converted to MCI within a mean of 13.1 months (Fernandez-Blazquez et al., 2016). While previous studies used the binary classification of conversion of CU to MCI, our study employed a data-driven classification to the CU based on longitudinal cognitive performance rather than on *a priori* classifications. A previous study using the mixture modeling showed that 6.7% of CU were classified into the declining group (Min, 2018). This discrepancy might be explained by the differences in neuropsychological test measurements between the previous study and our study. Previous studies used the MMSE (Small and Bäckman, 2007; Min, 2018), and this measure may not be sensitive enough to detect subtle cognitive changes in CU. However, our study used cognitive composites that are sensitive enough to detect subtle cognitive changes in CU, such as the PACC for the ADNI dataset and the LACPA for the SMC dataset. These two composite scores assess episodic memory, executive function, and global cognition (Donohue et al., 2014; Kim Y. J. et al., 2022) and have been frequently used to track cognitive changes in CU individuals (Donohue et al., 2014; Kim Y. J. et al., 2022).

Our second major finding was that increased Aβ uptake, decreased neuropsychological composite scores and decreased HV (only in ADNI) in individuals were predictive of being assigned to the declining group. Our findings are consistent with converging evidence. Specifically, a previous study suggested that cognitive performance, *APOE* ε4, decreased hippocampal and entorhinal cortex volumes, and increased cerebrospinal fluid p-tau were the most feasible predictors of CU to MCI conversion within 5 years (Albert et al., 2018). Therefore, the detection of these predictors in CU individuals has provided an important opportunity to understanding the contributions of these predictors to cognitive decline (Mormino and Papp, 2018).

Notably, there were some differences between the ADNI and SMC datasets with respect to the relative effects of each predictor. Specifically, relative to the ADNI dataset, the SMC dataset showed greater relative effects of females on the faster decliner. Previous studies indicated that females showed faster cognitive decline than males. Considering that the ADNI and SMC datasets include mainly non-Hispanic Whites (NHW) and Koreans, respectively, it might be related to the interactive effects of ethnicity and gender on cognitive decline. Although the underlying mechanisms are not fully understood, these gender- and ethnicity-specific differences might be related to genetic and sociocultural differences between Korean and NHW participants. In fact, a previous study from our group suggested that females suffered more deleterious effects of cardiometabolic syndrome on brain aging than males, which is more pronounced in Koreans than in UKs (Kang et al., 2022). Also, relative to the ADNI dataset, the SMC dataset showed greater relative effects of decreased neuropsychological composite scores than increased Aβ uptake and decreased HV. Further studies are needed to determine whether ethnicity influences Alzheimer’s pathology and progression.

Our final major finding was that predictive models of cognitive decline in CU individuals revealed fair to good discrimination and calibration capabilities. Previously, there were few predictive models for CU to MCI conversion. Nevertheless, previous studies have shown that the conversion of CU to MCI is influenced by fixed time and cognitive impairment levels. Our study’s contribution is the development of a model that can predict and visualize the individual risk of cognitive decline regardless of fixed time and set cognitive impairment levels. Furthermore, in the present study, visualization was performed by utilizing the nomogram to assess the predicted probability of a trajectory subgroup. As a result, our model may be able to produce clinically useful results allowing for a more clear interpretation. This model could facilitate identification of individuals who are at risk of cognitive decline.

The strength of the present study was that we developed predictive models based on two independent cohorts with multimodal imaging markers and by using a sophisticated GMM method. However, there are several issues that should be discussed. First, we did not include longitudinal changes in HV and Aβ in our model. Future studies are needed to determine whether incorporating longitudinal changes in HV and Aβ might increase prediction accuracy. In addition, since GMM estimates all variances, convergence errors can occur (McNeish et al., 2021). Nomograms assume that outcomes remain constant over time, and can become less accurate over time for a variety of reasons, such as improvements in earlier detection and management (Balachandran et al., 2015). Finally, other pathologies such as tau, argyrophilic grain disease, Lewy body disease, transactive response DNA-binding protein (TDP-43) pathology, hippocampal sclerosis, atherosclerosis, and gross infarcts that are associated with global cognitive decline were not considered (Wilson et al., 2020). Overall, despite these limitations, our study is unique in that it focuses on the cognitive trajectory of CU individuals and provides useful predictive models to identify CU individuals who may show future cognitive decline.

## Conclusion

In conclusion, our study provides novel insights into different cognitive trajectories among CU individuals. Since the predictive model was able to classify CU individuals with respect to potential future cognitive decline, this model could be employed in future primary AD prevention trials.

## Data availability statement

The raw data supporting the conclusions of this article will be made available by the authors, without undue reservation.

## Ethics statement

The studies involving human participants were reviewed and approved by the Institutional Review Board of Samsung Medical Center. The patients/participants provided their written informed consent to participate in this study.

## Author contributions

YK: conceptualization, formal analysis, methodology, resources, writing—original draft, and writing—review and editing. SK and AH: conceptualization, methodology, resources, writing—original draft, and writing—review and editing. HJ and JK: methodology, resources, and writing—review and editing. HK and DN: conceptualization, resources, and writing—review and editing. JC: conceptualization, resources, supervision, and writing—review and editing. SS: conceptualization, funding acquisition (lead), resources, supervision (lead), and writing—review and editing. All authors contributed to the article and approved the submitted version.

## Footnotes

1
www.adni-info.org

2
adni.loni.usc.edu

3
http://surfer.nmr.mgh.harvard.edu/

4
http://www.R-project.org/

## Supplementary material

Supplementary Figure 1 Bootstrap calibration curve for the ADNI **(A)** and SMC **(B)** models, showing the validation results in terms of discrimination capabilities. Dotted line, apparent calibration accuracy; solid line, bias-corrected calibration curve; and dashed line, ideal calibration. This model is well calibrated, because the bias-corrected calibration plot is close to the 45° line. ADNI, Alzheimer’s Disease Neuroimaging Initiative; SMC, Samsung Medical Center.
