# Supplementary figures and images for: Classification and prediction of cognitive trajectories of cognitively unimpaired individuals
Source: Front Aging Neurosci. 2023 Mar 13;15:1122927. doi: 10.3389/fnagi.2023.1122927 (PMC10040799; doi:10.3389/fnagi.2023.1122927)

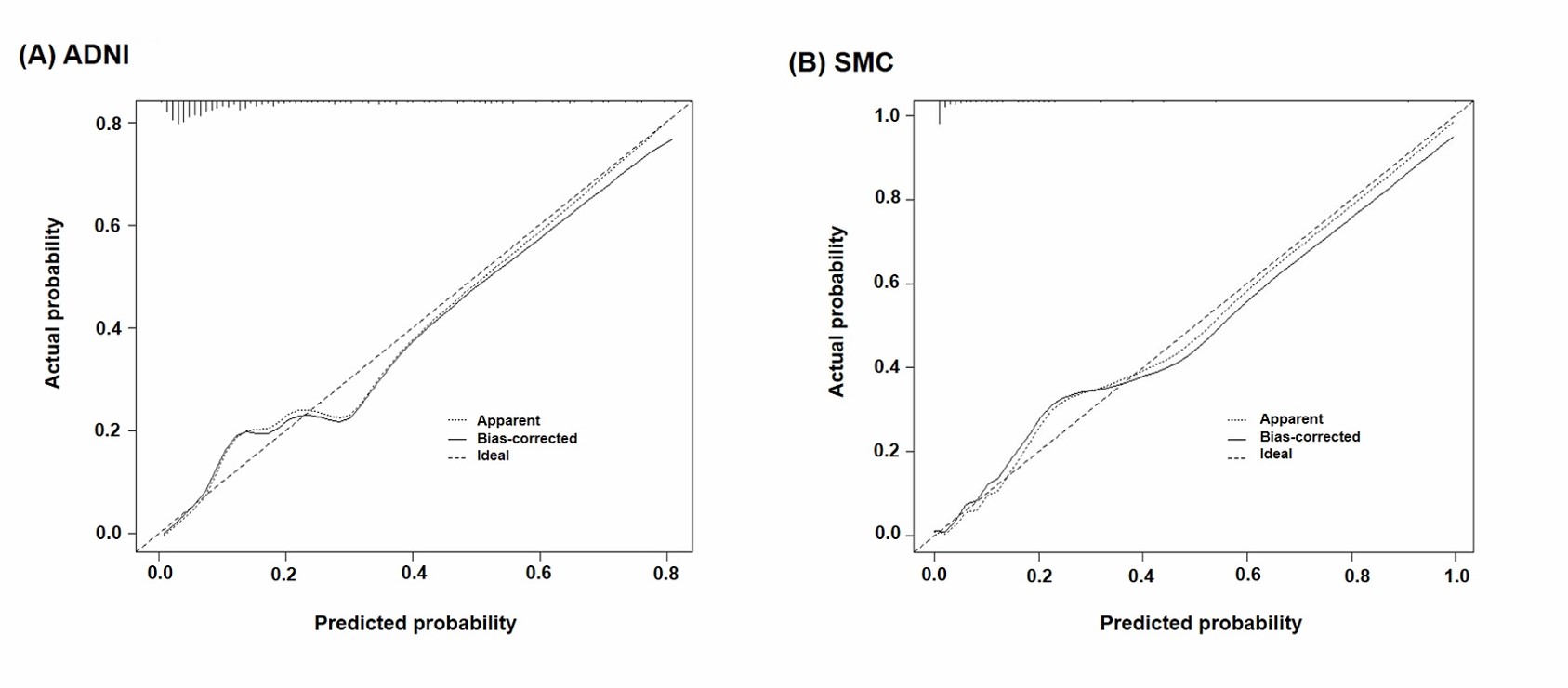

Supplement: Supplementary Figure 1 — Bootstrap calibration curve for the ADNI (A) and SMC (B) models, showing the validation results in terms of discrimination capabilities. Dotted line, apparent calibration accuracy; solid line, bias-corrected calibration curve; and dashed line, ideal calibration. This model is well calibrated, because the bias-corrected calibration plot is close to the 45° line. ADNI, Alzheimer’s Disease Neuroimaging Initiative; SMC, Samsung Medical Center. [file Image_1.JPEG]
